# MDN1 Mutation Is Associated With High Tumor Mutation Burden and Unfavorable Prognosis in Breast Cancer

**DOI:** 10.3389/fgene.2022.857836

**Published:** 2022-03-21

**Authors:** Shuai Hao, Miao Huang, Xiaofan Xu, Xulin Wang, Liqun Huo, Lu Wang, Jun Gu

**Affiliations:** ^1^ Department of General Surgery, Jinling Hospital, Medical School of Nanjing University, Nanjing, China; ^2^ Nursing School, Chongqing Medical University, Chongqing, China

**Keywords:** breast cancer, MDN1, tumor mutation burden, GSEA, TCGA

## Abstract

**Background:** Breast cancer (BRCA) is the most common cancer worldwide and a serious threat to human health. MDN1 mutations have been observed in several cancers. However, the associations of MDN1 mutation with tumor mutation burden (TMB) and prognosis of BRCA have not been investigated.

**Methods:** Genomic, transcriptomic, and clinical data of 973 patients with BRCA from The *Cancer* Genome Atlas (TCGA) database were analyzed. The clinical attributes of BRCA based on the MDN1 mutation status were assessed by comparing TMB and tumor infiltrating immune cells. Gene ontology analysis and gene set enrichment analysis (GSEA) were conducted to identify the key signaling pathways associated with MDN1 mutation. Moreover, univariate and multivariate Cox regression analyses were performed to assess the association between prognostic factors and survival outcomes. Finally, nomograms were used to determine the predictive value of MDN1 mutation on clinical outcomes in patients with BRCA.

**Results:** MDN1 was found to have a relatively high mutation rate (2.77%). Compared to the MDN1 wild-type patients, the TMB value was significantly higher in MDN1 mutant patients (*p* < 0.001). Prognostic analysis revealed that MDN1 mutant patients had a worse survival probability than MDN1 wild-type patients (hazard ratio = 2.91; 95% CI:1.07–7.92; *p* = 0.036). GSEA revealed samples with MDN1 mutation enriched in retinol metabolism, drug metabolism cytochrome P450, glucuronidation, miscellaneous transport, and binding event pathways.

**Conclusion:** MDN1 mutation was found to be associated with high TMB and inferior prognosis, suggesting that MDN1 mutation may play a potential role in prognosis prediction and immunotherapy guidance in BRCA.

## Introduction

According to the latest cancer statistics, breast cancer (BRCA) surpassed lung cancer as the leading cancer in the world, with 2.26 million new cases reported in 2020. BRCA is thus a serious threat to human health (Sung et al., 2021). Owing to the development of medical technology and genomics, malignant tumor has been gradually revealed to be caused by genetic mutation. The diagnosis and treatment of cancer have evolved from empirical medicine and evidence-based medicine to precision medicine (Liu et al., 2019). Despite receiving highly effective and comprehensive treatment, some patients develop drug resistance or recurrence and metastasis, which markedly affect their prognosis and survival. The molecular characteristic of BRCA determine the therapeutic response and prognosis (Dai et al., 2015). The innovation of genetic testing technology has paved the way for identifying new cancer biomarkers and therapeutic targets.

Ribosomes are important organelles responsible for human protein synthesis; however, their assembly and maturation are highly complex. Based on accumulating evidence, abnormalities in ribosomal biosynthesis can affect tumorigenesis (Pelletier et al., 2018). In addition to ribosomal RNA (rRNA) and ribosomal protein, more than 300 ribosomal assembly factors perform important regulatory function in ribosomal biosynthesis (de la Cruz et al., 2018). It is reported that ribosome export 7 (IRix7), ribosome export/assembly 1 (Rea1) and diazaborine resistance gene 1 (Drg1), which belong to the ATPase associated with diverse activities (AAA-ATPase) family, are associated with the formation of the large 60S subunit (Kressler et al., 2012).

MDN1, also known as Midasin AAA-ATPase 1, KIAA0301, and Real1, is a protein coding gene. MDN1 provides energy for the efficient removal of specific assembly factors from pre-60S particles after they have fulfilled their function (Prattes et al., 2019). Only a few studies have reported that the MDN1 mutation exists in carcinomas and may be associated with tumor oncogenesis and prognosis. For example, Liu et al. successfully identified two distinct mutation signature clusters (MSCs) featured by dominant somatic copy number alterations and massive mutations, respectively. MSC-1 displayed higher immunogenicity and immune infiltration, and among them, MDN1 was identified as a novel driver gene that could effectively distinguish different subtypes (Zaoqu Liu et al., 2021). Besides, Lobl et al. conducted a comprehensive synthesis and analysis of cutaneous squamous cell carcinoma (SCC) sequencing studies with individual clinical data. Based on their findings, MDN1 was found to be one of the mutations in more than 25% of SCCs, which indicates a potential applicability in targeted therapy. (Lobl et al., 2021). However, the relationship between MDN1 mutation and BRCA has not been reported to date. Therefore, the diagnostic and prognostic value of MDN1 mutation in BRCA require further assessment.

In this study, we aimed to analyze the mutation signature of MDN1 in BRCA based on TCGA and the influence of MDN1 mutation on TMB and immune infiltrating cells. MDN1 mutation-related biological pathways involved in BRCA were also detected using gene set enrichment analysis (GSEA), and a multivariate logistic regression based on the MDN1 mutation and clinicopathological parameters was established as a nomogram to predict BRCA prognosis.

## Materials and Methods

### Data Source

The “Masked Somatic Mutation” data were selected from the Genomic Data Commons (GDC) website (https://portal.gdc.cancer.gov/) to serve as the somatic mutation data for patients with BRCA (*n* = 986). The data were preprocessed using the VarScan software and the somatic mutations were visualized using the maftools R package (Mayakonda et al., 2018). The gene expression sequencing data (HTseq-Counts) of patients with BRCA (*n* = 1,092) were downloaded and the count values were converted to transcript per million (TPM) values. In addition, the clinical information of TCGA-BRCA matched patients (*n* = 1,097), including age, survival status, follow-up time, and TNM stage, was downloaded and assessed using GDC software. Data with no information on survival, estrogen receptor (ER), progesterone receptor (PR), or incomplete TNM staging were excluded. The final data from 959 patients were retained to generate clinical baseline information tables (Table 1).

**TABLE 1:** TCGA-BRCA patient baseline information (Note: The total number of patients with BRCA in this table is 959 and the total number of patients in the dataset is 1,085; this difference is due to a lack of clinical information such as PR, ER or TNM stage for some patients. Owing to the missing information, only 959 patients with complete information on the following characteristics were retained.)

Characteristic	Levels	Overall
N		959
age, n (%)	< 60	511 (53.3%)
	>=60	448 (46.7%)
PR, n (%)	Negative	321 (33.5%)
	Positive	638 (66.5%)
ER, *n* (%)	Negative	224 (23.4%)
	Positive	735 (76.6%)
T, *n* (%)	T1	245 (25.5%)
	T2	557 (58.1%)
	T3	126 (13.1%)
	T4	31 (3.2%)
N, *n* (%)	N0	448 (46.7%)
	N1	326 (34%)
	N2	106 (11.1%)
	N3	64 (6.7%)
	NX	15 (1.6%)
M, *n* (%)	M0	800 (83.4%)
	M1	17 (1.8%)
	MX	142 (14.8%)
Type, n (%)	MUT	27 (2.8%)
	WT	932 (97.2%)

### Copy Number Variation Analysis

To analyze the copy number variation of key genes in TCGA-BRCA patients, we downloaded the “Masked Copy Number Segment” data (*n* = 1,098) using TCGAbiolinks R package (Colaprico et al., 2016). “Segment_mean” values were obtained to reflect the amplification and deletion of copy number variations, where greater than 0 were amplification, and less than 0 were deletion. Besides, the information from the downloaded CNV segments was used to perform GISTIC 2.0 (Mermel et al., 2011) analysis in the GenePattern software (https://cloud.genepattern.org) (Coletta et al., 2012) to determine the copy number variation between the MDN1 mutation-type (MUT) and wild-type (WT) groups. “SNP6 GRCh38 Remapped Probeset File” were used as annotation.

### Tumor Mutation Burden Analysis

Tumor mutation burden (TMB) was defined as the total number of somatic mutations (except silent mutations) for each tumor sample (Merino et al., 2020). The TMB value was calculated for each sample by the “tmb” function of maftools R package, and the overall difference in TMB levels between the MDN1 MUT group and WT group was compared using the Wilcoxon signed rank test.

### Differentially Expressed Gene (DEG) Analysis

To determine the effect of MDN1 mutation on tumorigenesis in patients with BRCA, samples of patients in TCGA database were divided into MDN1 MUT and WT groups. Further, DEGs between the two groups were analyzed using the DESeq2 R package (Love et al., 2014) based on the “Count” value of patients, with the threshold setting of log2 (fold change) > 1.0 and *p* < 0.05. Heatmap and volcano plot were used to visualize overall distribution of DEGs.

### Functional Enrichment Analysis

Gene Ontology (GO) analysis is a common approach used in large-scale functional enrichment studies of biological process (BP), molecular function (MF), and cellular component (CC) (Ashburner et al., 2000). The Kyoto Encyclopedia of Genes and Genomes (KEGG) is a widely used database that stores information on genomes, biological pathways, diseases, and drugs (Ogata et al., 1999). GO annotation and KEGG pathway enrichment analyses of differential genes were performed using the cluster Profiler R package (Yu et al., 2012), with a critical value of FDR <0.05 considered statistical significant. Based on the results of the enrichment analysis, the results of GO and KEGG were visualized using the GO plot package (Walter et al., 2015). In addition, to investigate the differences in biological processes between different subgroups, a GSEA, which is a computational method to analyze whether a particular gene set is statistically different between two biological states, was performed. GSEA is commonly used to estimate changes in pathway and biological process activity in samples of expression datasets (Suarez-Farinas et al., 2010). The gene set “c2. cp.kegg.v7.2.-symbols” was downloaded from the MSigDB database (Liberzon et al., 2015) for GSEA, and FDR <0.25 and *p* < 0.05 were considered as significant enrichment.

### Association Between Gene Mutation and Tumor Immune Infiltration

The CIBERSORT tool (http://CIBERSORT.stanford.edu/) and the LM22 gene set were used to predict the proportion of 22 immune cell infiltrations in all samples of the dataset (Newman et al., 2015). We used the CIBERSORT package to assess the relative abundance of 22 immune cells in TCGA-BRCA dataset and calculate the correlation between them. We also analyzed the differential expression of HLA family genes in the MDN1 mutant and wild groups.

### Prognostic Model Generation and Prediction

First, a survival analysis of MDN1 gene between the MDN1 MUT and WT groups in TCGA-BRCA dataset was performed. Subsequently, the impact of MDN1 gene mutations combined with clinicopathological features on patient prognosis was investigated. Univariate and multivariable Cox regression analyses were used to identify independent prognostic factors. Subsequently, the MDN1 gene mutation information on TCGA-BRCA dataset patient and other clinical information were used to construct a nomogram for survival prediction, leading to personalized prognostic assessment of patients with BRCA. The riskscore formula was established according to multivariate Cox regression and the riskscore of each patient was calculated as following:

$$\text{risk}\mathrm{Score}\;=\sum_{i}\mathrm{Coefficient}\left(\mathrm{gen}e_{i}\right)*\mathrm{mRNA}\;\mathrm{Expression}\left(\mathrm{gen}e_{i}\right)$$

High- and low-riskscore group were divided according to the median riskscore of all patients.

### Statistical Analysis

All data processing and analysis were carried out using the R software (version 4.0.2). For the comparison of two groups of continuous variables, the statistical significance of normally distributed variables was estimated by independent the Student’s *t*-test and differences between non-normally distributed variables were analyzed by the Wilcoxon rank sum test. The chi-square test or Fisher’s exact test was used to compare and analyze the statistical significance between two groups of categorical variables. Univariate and multivariable Cox regression analyses were used to identify independent prognostic factors. Subject working characteristic receiver operating characteristic (ROC) curves were plotted, and the area under the curve (AUC) was calculated to assess the accuracy of the risk score in estimating prognosis. All statistical *p*-values were two-sided and statistically significant at *p* < 0.05.

## Results

### Analysis of the Overall Mutation Levels and Chromosomal Copy Number Variation in Patients With BRCA

**FIGURE 1:**
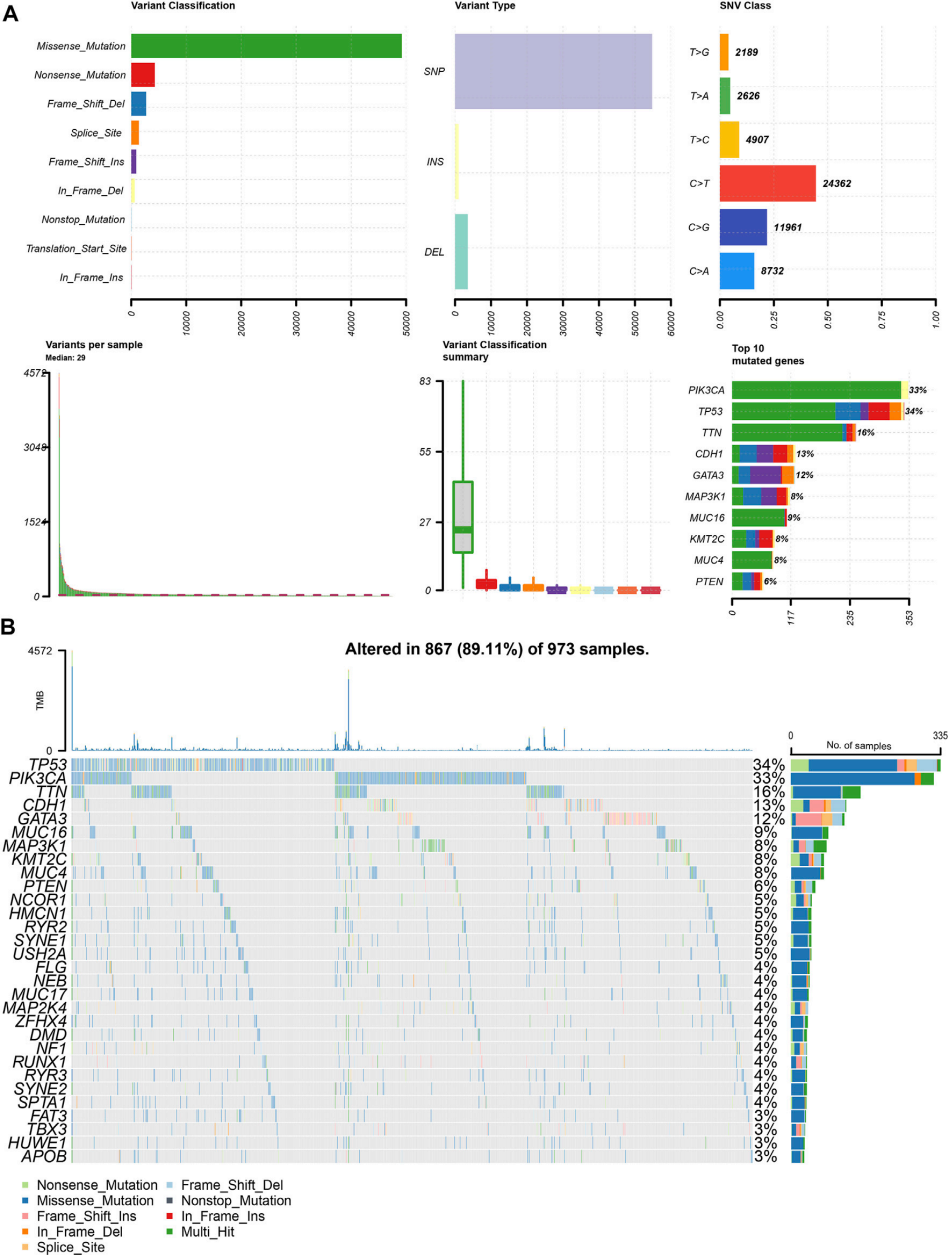
Panoramic view of TCGA-BRCA mutations. **(A)** Mutation panorama analysis of TCGA-BRCA dataset; **(B)** Top 30 gene mutation waterfall map of TCGA-BRCA dataset.

To determine the effect of mutations on patients with BRCA, we obtained mutation data of 973 patients after screening out samples with incomplete information from TCGA-BRCA dataset. First, we analyzed the overall mutation profile of TCGA-BRCA patients, which revealed that missense mutation accounted for the major portion, single nucleotide polymorphisms occurred more frequently than insertions or deletions, and C > T was the most common type of single nucleotide variants in patients with BRCA (Figure 1A). The top three mutated genes were *TP53, PIK3CA*, and *TTN* in all patients with BRCA (Figure 1B).

**FIGURE 2:**
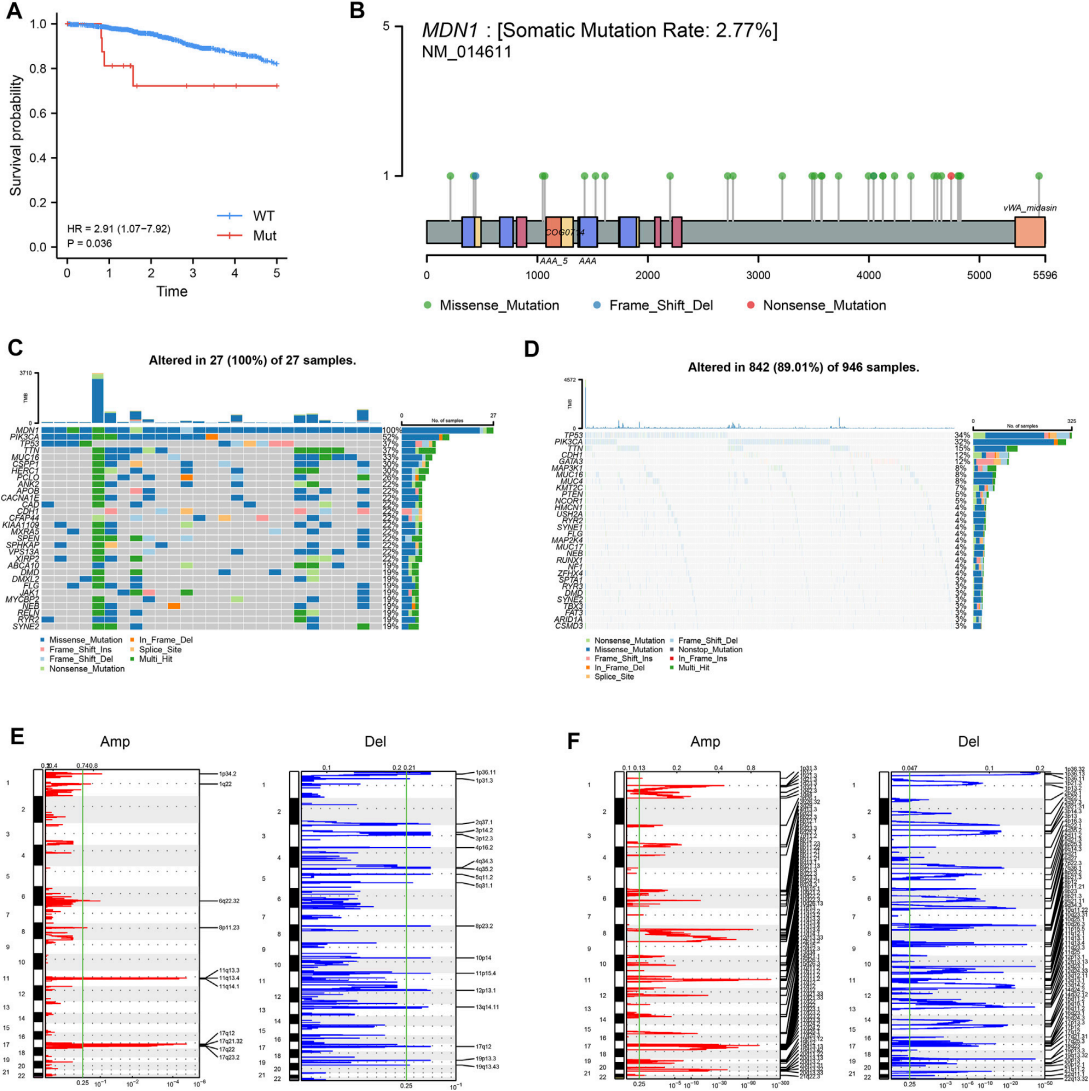
Analysis of mutation panorama and CNV changes in the MDN1 MUT group and WT group. **(A)** Prognostic analysis of MDN1 mutation status in TCGA-BRCA; **(B)** Information regarding the MDN1 mutation sites in TCGA-BRCA; **(C)** Mutation genes and types in the MDN1 MUT group in TCGA-BRCA; **(D)** Mutation genes and types in the MDN1 WT group in TCGA-BRCA; **(E-F)** Variation of the MDN1 copy number in mutation group and wild group; red represents gene amplification and blue represents gene deletion.

Based on the mutations in TCGA-BRCA, we analyzed the prognostic value of mutated genes with mutation frequencies >20 in the dataset and found that patients with mutated MDN1 had a significantly worse prognosis than those with WT MDN1 (Figure 2A). Based on the mutation status of MDN1, we divided the patients into MDN1 MUT group and MDN1 WT group, and used the lollipop plot and oncoplot plot to denote mutation sites of the MDN1 gene and the overall mutation level of the two groups of patients, respectively (Figures 2B–D). The main mutation form of the MDN1 protein was demonstrated to be concentrated in the missense mutation form. Further, the CNV levels of several genes were found to significantly change between the MDN1 mutation group and the WT group (Figures 2E,F).

### Biological Characterization of MDN1 Gene Mutation and Drug Sensitivity Analysis

**FIGURE 3:**
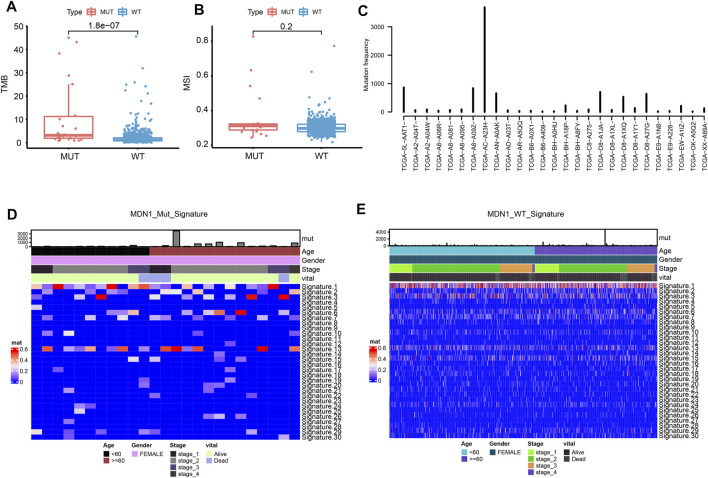
Biological characterization of MDN1 gene mutation in patients with BRCA. **(A)** TMB levels were significantly higher in patients with MDN1 mutation than in MDN1 wild-type patients (*p* < 0.001); **(B)** MSI values were not significantly different between the two groups (*p* = 0.2); **(C)** Individual mutation frequencies in MDN1 mutated group; **(D)** COSMIC signature heatmap analysis of MDN1 mutated patients from TCGA-BRCA dataset; **(E)** COSMIC signature heatmap analysis of patients in the MDN1 WT group from TCGA-BRCA dataset.

We further analyzed the correlation between MDN1 mutation and different biological characteristics. TMB was found to be significantly higher in the MDN1 MUT group than in the WT group (*p* < 0.001), while the microsatellite instability (MSI) did not significantly differ between the two groups (*p* = 0.2) (Figures 3A,B). According to the mutation frequency, we analyzed the mutations of individual patients in the MDN1 MUT group and found that they were significantly higher in patient “TCGA-AC-A23H” than in other patients (Figure 3C). Sanger et al. [https://software.broadinstitute.org/cancer/cga/msp] decomposed the 96-mutation spectrum into 30 different signatures based on somatic mutation characteristics combined with biological characteristics. We found that some signatures were significantly altered between the MDN1 MUT and WT groups (Figures 3D,E).

**FIGURE 4:**
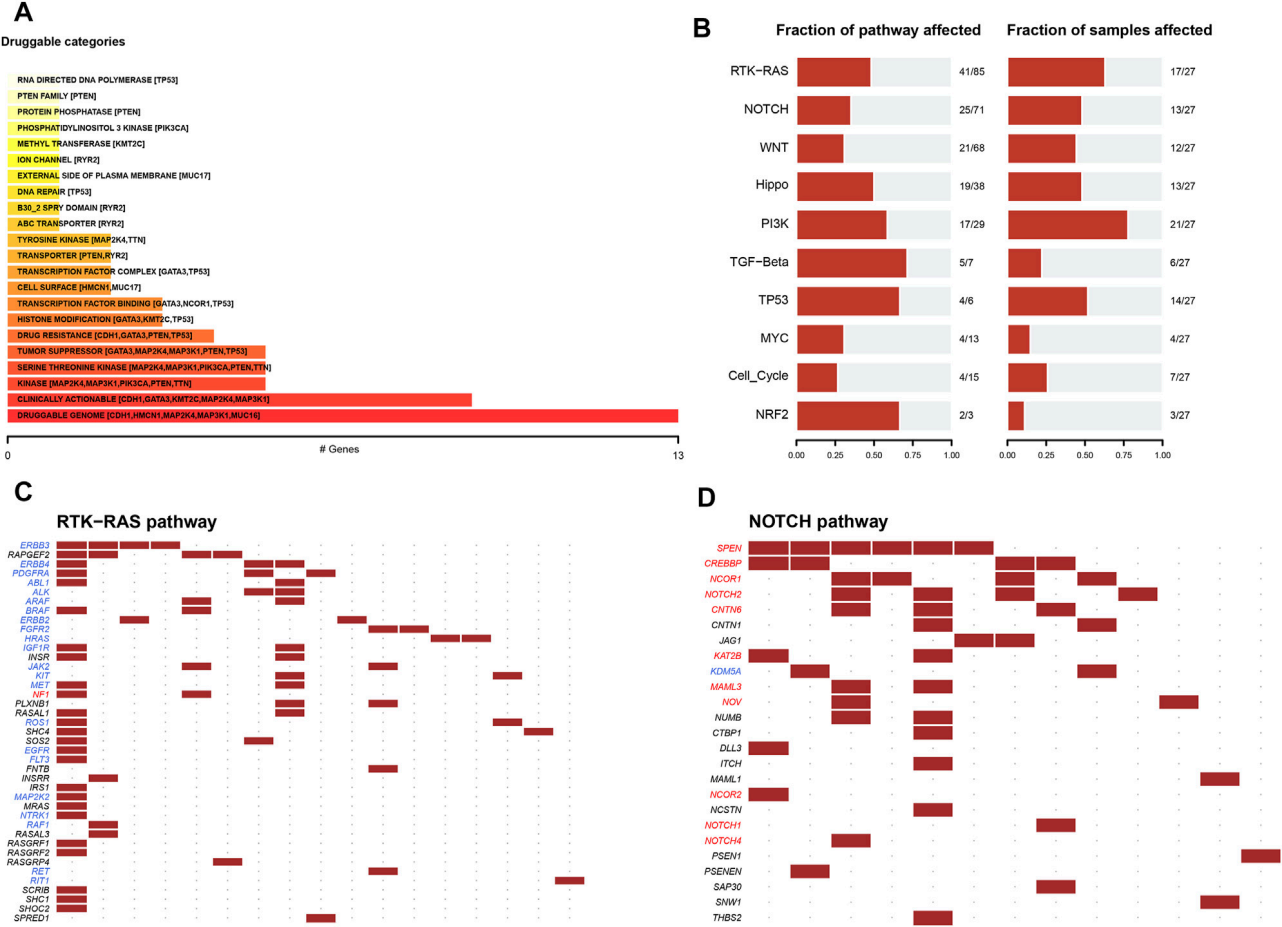
Drug sensitivity analysis of MDN1 gene mutations. **(A)** Overall analysis of the relationship between gene mutation levels and gene druggability information compiled from the drug-gene interaction database; **(B)** Analysis of the changes in gene mutation levels in different oncogenic signaling pathways in TCGA-BRCA dataset; **(C,D)** Distribution of gene mutations in the RTK-Ras and NOTCH signaling pathways in patients of TCGA-BRCA dataset.

When the influence of gene mutation on pathway and drug sensitivity was evaluated, multiple drug classes were found to be correlated with the mutation status of the MDN1 gene in TCGA-BRCA patients, especially with the druggable genome (Figure 4A). Oncogenic signaling pathway analysis showed that RTK-Ras pathway, NOTCH pathway, and WNT pathway were significantly enriched in the MDN1 MUT group (Figure 4B). The mutations of related genes in the RTK-Ras pathway and NOTCH pathway are depicted using waterfall plots (Figures 4C,D).

### Functional Enrichment in Patients With MDN1 Mutation

**FIGURE 5:**
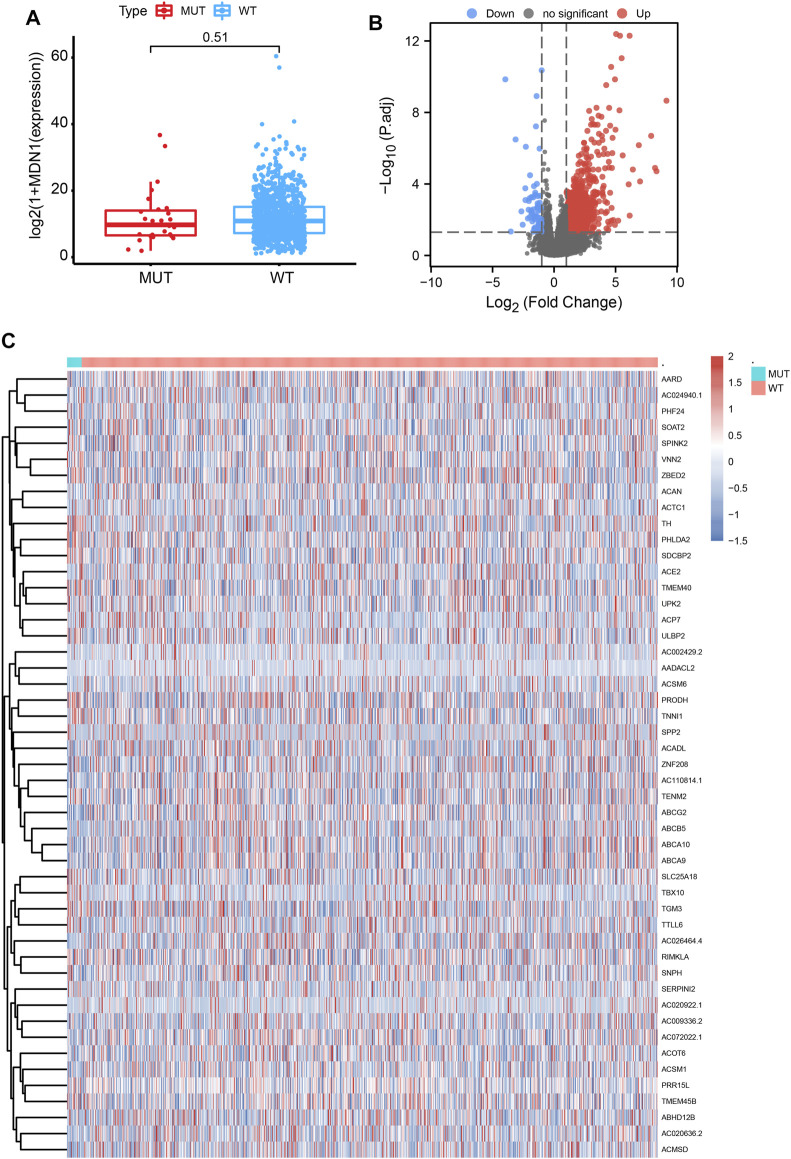
Differentially expressed genes (DEGs) based on the mutation status of the MDN1 gene. **(A)** No significant difference in MDN1 expression levels was found in the MDN1 mutation group compared to the MDN1 wild-type group (*p* = 0.51); **(B,C)** Volcano and heatmaps showing the expression of DEGs between the MDN1 mutation group and MDN1 wild-type group.

To analyze the effect of MDN1 gene mutation on tumorigenesis in TCGA-BRCA, patients were divided into MDN1 MUT group and WT group. No significant difference was found in MDN1 gene expression levels between the two groups (*p* = 0.51, Figure 5A). Subsequently, a DEG analysis was performed between the two groups. Based on the results, 910 genes were significantly upregulated and 77 genes were significantly downregulated in the MDN1-MUT group. We selected the top 25 upregulated and downregulated genes to plot heatmaps (Figures 5B,C).

**FIGURE 6:**
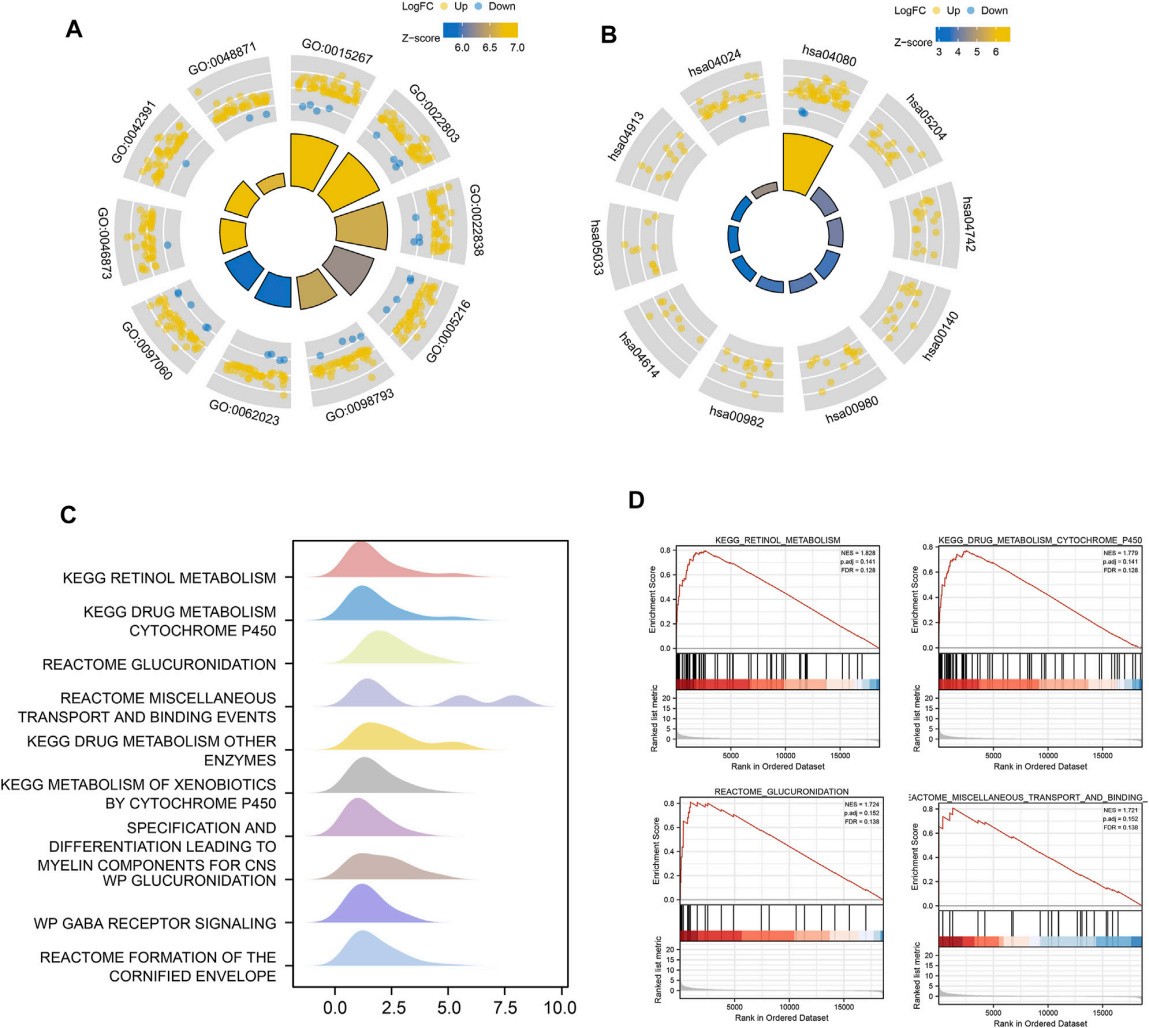
Functional enrichment analysis of DEGs based on MDN1 gene mutation. **(A)** The circle diagram shows the GO term where DEGs are mainly enriched; **(B)** The circle diagram shows the KEGG pathway where DEGs are mainly enriched; **(C)** GSEA mountain range plot showing the top 10 GSEA enriched pathways according to NES; **(D)** Top four GSEA enriched pathways. NES value represents the normalized enrichment score; the larger the NES value, higher the number of genes enriched in that pathway; *p*-value reflects the confidence of the enrichment results.

Based on GO term enrichment findings, the DEGs were closely associated with GO:0015267 (channel activity), GO:0022803 (passive transmembrane transporter activity), GO:0022838 (substrate-specific channel activity), and GO:0098793 (presynapse) (Figure 6A). KEGG pathway enrichment analysis revealed that these DEGs mainly affected the hsa04080 (Neuroactive ligand-receptor interaction), hsa04024 (cAMP signaling pathway), and hsa04020 (Calcium signaling pathway) pathways (Figure 6B) (Table 2). We analyze the functional enrichment pathways of the MDN1 MUT and MDN1 WT groups in TCGA-BRCA dataset by GSEA (Table 3). Retinol metabolism, drug metabolism cytochrome P450, glucuronidation, miscellaneous transport and binding events, and other pathways were found to be significantly enriched in the MDN1 MUT group (Figures 6C,D **)**.

**TABLE 2:** GO and KEGG analysis of DEGs in the MDN1 mutant and wild-type groups.

Type	Id	Description	Count	p.adjust
MF	GO:0015267	channel activity	61	8.08E-12
MF	GO:0022803	passive transmembrane transporter activity	61	8.08E-12
MF	GO:0022838	substrate-specific channel activity	58	1.24E-11
CC	GO:0098793	presynapse	57	7.49E-09
MF	GO:0005216	ion channel activity	54	3.27E-10
BP	GO:0042391	regulation of membrane potential	53	1.51E-07
BP	GO:0048871	multicellular organismal homeostasis	52	5.96E-06
CC	GO:0097060	synaptic membrane	50	5.00E-08
MF	GO:0046873	metal ion transmembrane transporter activity	50	8.87E-08
CC	GO:0062023	collagen-containing extracellular matrix	49	2.05E-08
CC	GO:0043025	neuronal cell body	49	8.12E-06
MF	GO:0022836	gated channel activity	49	1.25E-10
BP	GO:0050804	modulation of chemical synaptic transmission	48	6.29E-06
BP	GO:0099177	regulation of trans-synaptic signaling	48	6.29E-06
MF	GO:0022839	ion gated channel activity	47	4.30E-10
BP	GO:0072503	cellular divalent inorganic cation homeostasis	46	3.49E-04
BP	GO:0023061	signal release	45	1.94E-04
MF	GO:0005261	cation channel activity	45	9.86E-10
MF	GO:0048018	receptor ligand activity	45	6.94E-05
BP	GO:0006874	cellular calcium ion homeostasis	44	3.12E-04
KEGG	hsa04080	Neuroactive ligand-receptor interaction	57	2.88E-15
KEGG	hsa04024	cAMP signaling pathway	25	6.80E-04
KEGG	hsa04020	Calcium signaling pathway	20	1.16E-02
KEGG	hsa05204	Chemical carcinogenesis	17	2.41E-05
KEGG	hsa04742	Taste transduction	17	3.31E-05
KEGG	hsa04724	Glutamatergic synapse	16	1.28E-03
KEGG	hsa04530	Tight junction	16	4.50E-02
KEGG	hsa00980	Metabolism of xenobiotics by cytochrome P450	15	1.18E-04
KEGG	hsa04713	Circadian entrainment	15	9.93E-04
KEGG	hsa04726	Serotonergic synapse	15	3.82E-03
KEGG	hsa00140	Steroid hormone biosynthesis	14	3.98E-05
KEGG	hsa00982	Drug metabolism - cytochrome P450	14	1.84E-04
KEGG	hsa04512	ECM-receptor interaction	14	1.13E-03
KEGG	hsa04974	Protein digestion and absorption	14	3.82E-03
KEGG	hsa05414	Dilated cardiomyopathy	13	6.16E-03
KEGG	hsa05031	Amphetamine addiction	12	1.28E-03
KEGG	hsa05412	Arrhythmogenic right ventricular cardiomyopathy	12	3.28E-03
KEGG	hsa04976	Bile secretion	12	9.82E-03
KEGG	hsa04972	Pancreatic secretion	12	2.60E-02
KEGG	hsa04913	Ovarian steroidogenesis	11	5.44E-04

**TABLE 3:** GSEA of DEGs in the MDN1 mutant and wild-type groups.

Description	setSize	NES	Pvalue
KEGG_RETINOL_METABOLISM	64	1.83	1.01E-03
KEGG_DRUG_METABOLISM_CYTOCHROME_P450	70	1.78	1.01E-03
REACTOME_GLUCURONIDATION	25	1.72	2.16E-03
REACTOME_MISCELLANEOUS_TRANSPORT_AND_BINDING_EVENTS	25	1.72	2.16E-03
KEGG_DRUG_METABOLISM_OTHER_ENZYMES	51	1.72	1.02E-03
KEGG_METABOLISM_OF_XENOBIOTICS_BY_CYTOCHROME_P450	68	1.71	1.01E-03
WP_OLIGODENDROCYTE_SPECIFICATION_AND_DIFFERENTIATION_LEADING_TO_MYELIN_COMPONENTS_FOR_CNS	29	1.70	2.14E-03
WP_GLUCURONIDATION	26	1.68	2.16E-03
WP_GABA_RECEPTOR_SIGNALING	31	1.67	2.11E-03
REACTOME_FORMATION_OF_THE_CORNIFIED_ENVELOPE	129	1.67	1.00E-03
REACTOME_INCRETIN_SYNTHESIS_SECRETION_AND_INACTIVATION	23	1.67	2.21E-03
REACTOME_XENOBIOTICS	23	1.67	2.21E-03
WP_OXIDATION_BY_CYTOCHROME_P450	61	1.65	1.01E-03
REACTOME_CYP2E1_REACTIONS	11	1.65	1.23E-03
KEGG_STEROID_HORMONE_BIOSYNTHESIS	55	1.65	1.02E-03
KEGG_PENTOSE_AND_GLUCURONATE_INTERCONVERSIONS	28	1.64	2.15E-03
KEGG_PORPHYRIN_AND_CHLOROPHYLL_METABOLISM	40	1.64	2.07E-03
KEGG_ASCORBATE_AND_ALDARATE_METABOLISM	25	1.64	2.16E-03
REACTOME_NUCLEAR_SIGNALING_BY_ERBB4	32	1.63	2.11E-03
KEGG_TASTE_TRANSDUCTION	51	1.62	2.04E-03

### Effect of MDN1 Gene Mutation on Immune Cell Infiltration

**FIGURE 7:**
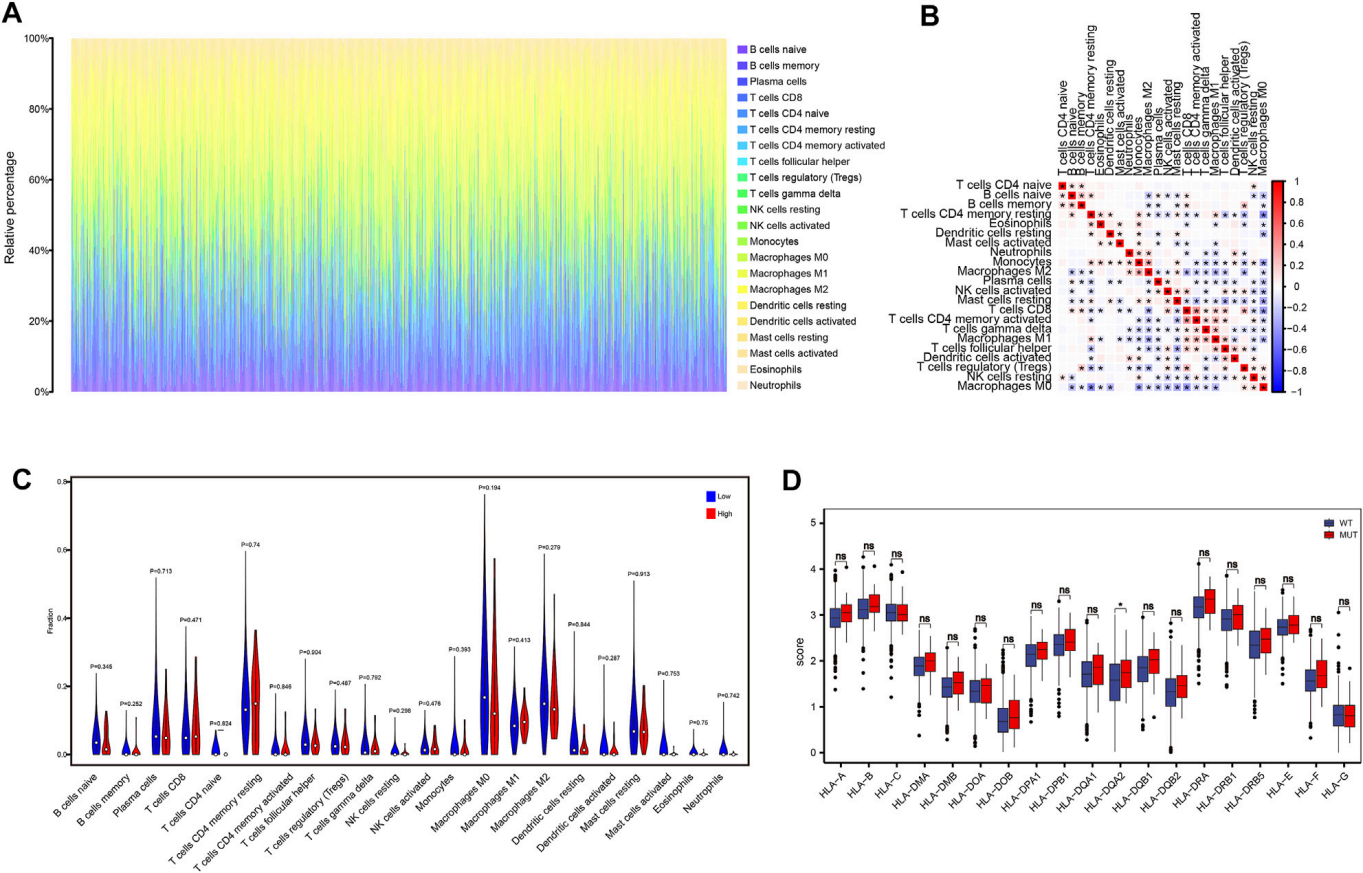
Effect of MDN1 gene mutation on immune cell infiltration in TCGA-BRCA dataset. **(A)** Panorama of immune cell infiltration in TCGA-BRCA dataset; **(B)** Correlation analysis of 22 immune cell types in TCGA-BRCA dataset; **(C)** Multiple immune cell infiltration levels do not differ significantly between different mutation groups of MDN1 gene in TCGA-BRCA patients; **(D)** HLA family genes are significantly different between the MDN1 mutation group and MDN1 wild-type group.

To analyze the relationship between MDN1 gene mutation and immune cell infiltration in the tumor microenvironment of the patients, we calculated the percentage of immune cell infiltration in the tumor microenvironment using the CIBERSORT algorithm. Figure 7A and Figure 7B show the panorama and correlation of immune cell infiltration in the tumor microenvironment, respectively. A few immune cells were found to differ between the MDN1 MUT group and MDN1 WT group; however, none of the 22 immune cell differences were statistically significant (Figure 7C). The expression of HLA family genes in the MDN1 MUT group versus the MDN1 WT group was also analyzed. As shown in Figure 7D, the expression levels of MHC class I genes, such as *HLA-A*, *HLA-B*, and *HLA-C*, were not statistically different between the two groups, whereas those of MHC class II genes, such as *HLA-DQA2*, were significantly different.

### Survival Analysis of MDN1 Gene Mutations Combined With Clinicopathologic Characteristics

**FIGURE 8:**
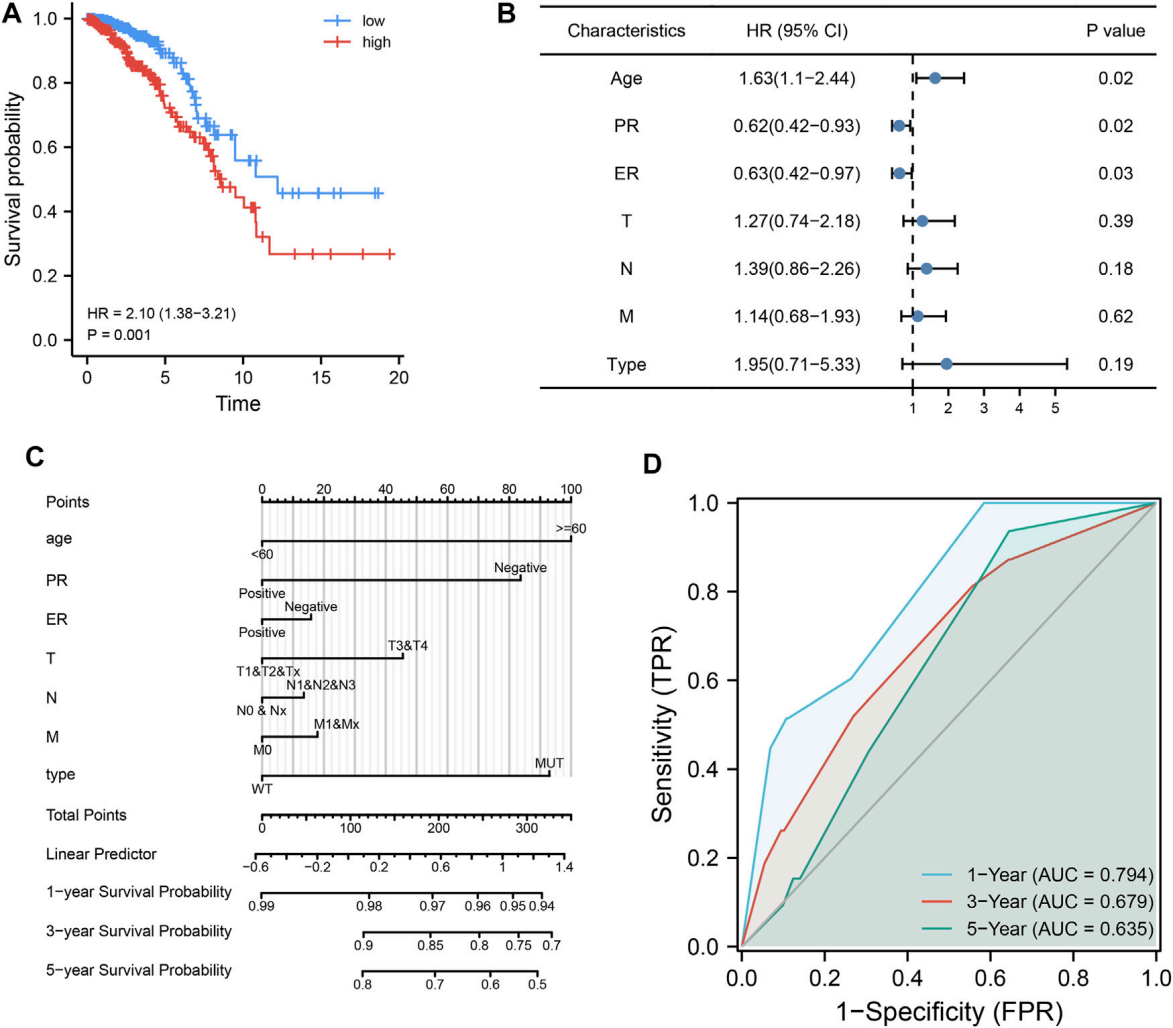
Effect of MDN1 gene mutation on the clinical prognosis in TCGA-BRCA dataset. **(A)** Cox regression analysis showed that the prognosis of patients with BRCA in the high-risk group was significantly worse (*p* = 0.001); **(B)** Forest plot showing the effect of univariate analysis of MDN1 gene mutations and different clinicopathological features on OS of patients; **(C)** MDN1 mutation combined with clinicopathological features to construct nomogram; **(D)** ROC curves for the model with area under the curve (AUC) of 0.794, 0.679, and 0.635 at 1, 3, and 5 years, respectively.

To further explore the impact of MDN1 mutations on TCGA-BRCA patients, a prognostic model was established using univariate and multivariate Cox regression analysis in combination with the clinicopathological characteristics of patients. The risk score of each sample obtained by multivariate analysis was ranked and divided into high- and low-risk groups by the median value. The prognosis of patients between the high- and low-risk group was significantly different, and the prognosis of patients in the high-risk group was worse (Figure 8A). Moreover, age, TNM stage, and MDN1 mutation status were identified as risk factors for patients with BRCA (HR > 1); however, the mutation status of the MDN1 gene was not statistically significant (*p* = 0.19) (Figure 8B). Subsequently, we incorporated MDN1 mutation combined with clinicopathological features into the model and constructed a nomogram to predict OS in patients with BRCA (Figure 8C). Further, ROC curves were used to validate the efficiency of the model and area under the curve (AUC) values of 0.794, 0.679, and 0.635 were determined for 1-, 3-, and 5-years OS, respectively (Figure 8D).

## Discussion

BRCA is the most common malignant tumor and the second leading cause of death due to cancer in women, thereby posing a serious threat to human health (Sung et al., 2021). The role of somatic mutations in cancer development and progression has been confirmed with the innovation of genetic testing technology, which brings new hope for identifying novel cancer markers and therapeutic targets. To our knowledge, the function and potential prognostic impact of MDN1 mutation on BRCA have not been investigated.

The protein encoded by the *MDN1* gene is one of the largest proteins in the body with a relative molecular mass of 632,000 and containing 5,596 amino acid residues. It is a member of the AAA-ATPases family, which plays an important role in pre-60S ribosome maturation. Abnormalities in ribosome biosynthesis, including ribosomal RNA instability, protein mutations, and assembly factor abnormities, might induce tumorigenesis and progression. As a result, these factors have potential applicability as therapeutic targets for a variety of tumors, such as colorectal cancer (Tsoi et al., 2017), cervical cancer (Zhou et al., 2016), and thyroid cancer (Guimaraes and Zavolan, 2016). As an important protein enzyme affecting the process of ribosome synthesis, MDN1 has been found to be mutated in a few cancers, such as colorectal cancer (Zaoqu Liu et al., 2021) and SCC of the skin (Lobl et al., 2021), and may act as a driver gene to promote the occurrence and development of cancer. In BRCA, MDN1 has a high mutation rate of 2.77%. However, the dysfunction of MDN1, especially its mutation, has rarely been studied in BRCA. Our result provided a foundation for the research of MDN1 mutation in BRCA for the first time.

First, we comprehensively analyzed the mutational signature of MDN1 in BRCA, in which missense mutation constitutes the main mutation type. Besides, we observed that the CNV levels of several genes were changed significantly between the MDN1 MUT and MDN1 WT groups. In addition, based on survival analysis, patients in the MDN1 MUT group were found to have a worse prognosis. Accordingly, we sought to further explore the underlying mechanisms.

Somatic mutations might affect the expression of downstream genes, leading to alterations in the transcriptional network that affect different phenotypes and functions. To determine the effect of MDN1 mutations on patients with BRCA, we performed transcriptome analysis and found that 910 genes were significantly upregulated and 77 genes were significantly downregulated in the MDN1 MUT group in TCGA-BRCA dataset. We respectively selected the top 25 upregulated and downregulated genes to plot a heatmap.

Proline dehydrogenase (PRODH) overexpression was identified in the MDN1 MUT group relative to the WT group. PRODH can catalyze the first step of proline catabolism and has diverse functional roles in regulating pathophysiological processes, including apoptosis, autophagy, cell senescence, and tumorigenesis. In many types of cancer, PRODH acts as oncogene to promote cancer occurrence and progression (Yating Liu et al., 2021). Elia et al. found that the upregulation of PRODH supported 3D growth and metastasis, generated ATP, and acted as a drug target in MCF10A BRCA cells (Elia et al., 2017). These findings confirmed the potential role of PRODH expression as a prognostic marker for BRCA. However, the relationship between MDN1 and PRODH remains to be evaluated. Another upregulated gene, *PHLDA2*, belongs to the pleckstrin homology like domain, family A (PHLDA) gene family and encodes protein that can inhibit AKT signaling through phosphoinositol binding competition. Mangone et al. found that breast tumor samples showed higher levels of PHLDA2 transcripts (*p* < 0.0001) than normal tissue samples. Besides, PHLDA2 could act as a prognostic marker and predict a response to endocrine therapy in BRCA (R Mangone et al., 2020). Moon et al. also concluded that the expression of PHLDA was high in triple negative BRCA cells (Moon et al., 2015).

Some members of the ABC family are downregulated in MDN1 mutation patients, such as ABCG2, ABCB5, ABCA9, and ABCA10. These proteins are alternatively referred to as BRCA resistance proteins and function as a xenobiotic transporter, which may play a major role in multi-drug resistance (Hlavac et al., 2020). Similarly, SNPH is downregulated, which suppresses metastasis by inhibiting the movement of mitochondria in cancer cells (Seo et al., 2018). These results indicated that the MDN1 mutation may influence BRCA drug resistance and induce tumor progression.

Concerning the DEGs, GSEA revealed samples with MDN1 mutation mainly enriched in “retinol metabolism, drug metabolism cytochrome P450, glucuronidation, miscellaneous transport and binding events pathways.” Among them, glucuronidation is the major pathway in phase II metabolism and accounts for approximately 35% of drug conjugation (Wells et al., 2004). Glucuronidation conjugation utilizes UDP-glucuronosyltransferases to catalyze a wide range of diverse endogenous and xenobiotic compounds. Zhou et al. found that UGT contributes to estrogen elimination, and its glucuronidation capacity influences the estrogen signaling pathway and pathogenesis of BRCA (Zhou et al., 2017). Similarly, the cytochrome P450 (P450) enzymes constitute the predominant enzyme system involved in human drug metabolism (Tracy et al., 2016), which plays a multi-faceted role in contributing to carcinogenesis, tumor growth, invasion and metastasis, especially in BRCA (Luo et al., 2021).

Whether MDN1 mutations promote BRCA development by affecting the enrichment of drug metabolism-related pathways is unclear. Further in-depth analysis using a drug-gene interaction database revealed that multiple drug classes correlated with MDN1 gene mutation, especially with the druggable genome. Oncogenic signaling pathways indicated that RTK-Ras pathway, NOTCH pathway, and WNT pathway were significantly enriched in patients with BRCA, suggesting that patients with MDN1 mutation had potential targets for prevention and therapy.

Immunotherapy based on immune checkpoint inhibitors has significantly improved the survival prognosis of patients with malignancies; however, BRCA is mostly considered to be a weakly immunogenic tumor type, which limits the effectiveness of immunotherapy. As a result, identifying patients that can benefit from immunotherapy and discovering reliable biomarkers are important in devising novel treatment strategies. (Savas et al., 2016). We found that patients in the MDN1 MUT groups had significantly increased levels of TMB compared to those in the MDN1 WT group. TMB, which generally refers to the number of nonsynonymous mutations per megabase pair (Mb) of somatic cells within a specific genomic region, has been shown to predict the effectiveness of immunotherapy for a variety of tumors (Chalmers et al., 2017). Patients with MDN1 mutations had potentially higher therapeutic benefit with immunotherapy. The analysis of immune components in the tumor microenvironment could provide insight into tumorigenesis and progression. For example, in the BRCA tumor microenvironment, tumor infiltrating lymphocyte (TIL) can respond to the treatment effect and prognosis of multiple BRCA subtypes and serve as a potential biomarker (Denkert et al., 2018).

We then analyzed the relationship between MDN1 gene mutation and immune cell infiltration in the BRCA microenvironment. Although some immune cells showed differences in different subgroups, none of the 22 immune cells showed significant differences. We further analyzed the expression of HLA family genes in the MDN1 MUT group versus the MDN1 WT group. MHC class I genes, such as *HLA-A*, *HLA-B*, and *HLA-C*, were not statistically different in the mutant group versus the wild group; however, significant differences were found in the expression of MHC class II genes, such as *HLA-DQA2*. HLA-DQA2 was found to play a central role in the peptide loading of MHC class II molecules by aiding the release of the CLIP molecule from the peptide binding site, which influences the MHC class II receptor activity (Lenormand et al., 2012). In conclusion, MDN1 mutation could increase TMB levels and promote MHC-II activity, thereby potentially enhancing the immunotherapeutic effects.

To further explore the impact of MDN1 gene mutation on the prognosis of TCGA-BRCA patients, prognostic models were established using univariate and multivariate Cox regression analysis in combination with the clinicopathological characteristics of patients. As a result, age, TNM stage, and MDN1 mutation-type were identified as risk factors for patients with BRCA (HR > 1). Based on the results, a nomogram was developed to predict the 1-, 3-, and 5- survival probabilities of patients with BRCA. Further, a ROC curve was used to determine the predictive validity of the nomogram to prospective outcomes, which revealed AUC values of 0.794, 0.679, and 0.635 for 1-year, 3-years, and 5-years OS, respectively.

Although our approach improved the understanding of the relationship between MDN1 mutation and BRCA, this study has some limitations. First, all data were obtained using online databases, although similar sample setting from TCGA database can be found in many studies (Wang et al., 2020; Zhang and Zhang, 2020; <u>Li et al., 2021), only one database was adopted as data source may cause sample bias. Second, despite one tumor “TCGA-AC-A23H” had several more mutations relative to the rest of the samples in the MDN1-MUT cohort. The current limited number of sample cases may also cause bias in the results if the individual mutation is excluded. In addition, other similar studies have included the entire sample and did not perform sample exclusion (Zhang and Zhang, 2020; Guo et al., 2021). Third, even several of the recently published high-scoring articles were analyzed based exclusively on the TCGA or/and GEO public databases, and all of these studies have implications for future clinical research (Hong et al., 2020; Yi et al., 2020; Guo et al., 2021). The functional pathway analysis of MDN1 mutations needs to be verified *in vitro* and *in vivo*. Besides, further experimental verifications are necessary to elucidate the detailed and direct mechanisms of the correlation between MDN1 mutation and BRCA.

## Conclusion

In conclusion, through the analysis of somatic mutation data in TCGA database, we found that MDN1 was a high frequency mutation gene in BRCA. Further, DEGs and pathway enrichment analysis revealed that MDN1 mutation affected multiple biological functions, including drug metabolism-related genes. MDN1 mutation was closely correlated with high TMB and MHC class II receptor activity, suggesting that it might be a potential therapeutic target and could contribute to the development of immunotherapy for BRCA. Furthermore, MDN1 mutation was an unfavorable prognostic biomarker and revealed the potential mechanism of MDN1 mutation on BRCA.

## Data Availability

The original contributions presented in the study are included in the article/Supplementary Material, further inquiries can be directed to the corresponding author.
